# Deep learning facilitates efficient optimization of antisense oligonucleotide drugs

**DOI:** 10.1016/j.omtn.2024.102208

**Published:** 2024-05-16

**Authors:** Shenggeng Lin, Liang Hong, Dong-Qing Wei, Yi Xiong

**Affiliations:** 1 State Key Laboratory of Microbial Metabolism, School of Life Sciences and Biotechnology, Shanghai Jiao Tong University, Shanghai, China; 2 Artificial Intelligence Biomedical Center, Zhangjiang Institute for Advanced Study, Shanghai Jiao Tong University, Shanghai, China; 3 Shanghai Artificial Intelligence Laboratory, Shanghai, China; 4 Institute of Natural Sciences, Shanghai Jiao Tong University, Shanghai, China

## Main text

Nucleic acid therapeutics exert their therapeutic effects by targeting specific genes through complementary sequence recognition to inhibit the expression of certain proteins or facilitate the translation and synthesis of proteins encoded by target genes.^1^ Nucleic acid drugs mainly include messenger RNA (mRNA) drugs and oligonucleotide drugs, which consist of antisense oligonucleotides (ASOs), small interfering RNAs (siRNAs), and RNA aptamers.^2^ mRNA vaccines have demonstrated high efficacy and reliability during the COVID-19 pandemic, while oligonucleotide drugs have shown tremendous promise in the treatment of genetic diseases.^3^ Up to present, the US Food and Drug Administration has approved a total of 17 RNA drugs or vaccines, including 9 ASO drugs, 5 siRNA drugs, 1 RNA aptamer drug, and 2 mRNA drugs, with 11 RNA drugs indicated for genetic diseases.^4^ The ASO drugs among these approved pharmaceuticals, exceeding 50%, underscore their efficacy and promising prospects. However, the application of ASO drugs also faces challenges, including poor stability, short half-life, immunogenicity, low targeted delivery efficiency, and safety issues.^3^ To ensure the safety and efficacy of ASO drugs, researchers have been working on reducing “off-target effects” and chemically modifying nucleotides to minimize side effects.^5^

Traditionally, researchers have heavily relied on their molecular biology expertise and empirical observations to generate potential ASO candidates. However, the intrinsic complexity of nucleic acid mechanisms coupled with the vast search space of nucleic acid sequences necessitates a paradigm shift toward computational methods.^6^ Previous efforts to optimize nucleic acid drugs using computational methods have mainly focused on siRNAs and mRNAs, leaving challenges in optimizing ASO drugs largely unresolved.^7^ In recent years, deep learning has shown great promise in optimizing biological sequences, particularly in protein sequence optimization, offering hope for addressing the challenges in ASO drug optimization.^8^ Ryu and colleagues developed the deep learning-based platform ASOptimizer, which can efficiently design ASOs targeting IDO1 mRNA.^6^ Experimental results demonstrate the effectiveness of the ASOptimizer platform in predicting and optimizing ASOs. The major limitation of the study is the lack of validation on wider range of cases beyond instances targeting IDO1 gene regulation due to insufficient data.

ASOptimizer incorporates two key components: sequence engineering and chemical engineering. At the sequence engineering stage, ASOptimizer optimizes and predicts ASO sequences that efficiently target IDO1 mRNA. ASOptimizer utilizes a linear factor model that correlates the regulatory efficiency of ASOs with their thermodynamic properties and secondary structures. The linear model was trained on a subset of the comprehensive database, which consists of experimental inhibition rates specifically targeting the IDO1 gene. After training, the model sorted 19-base complementary ASOs and selected six top candidates for further experimental validation *in vitro*. The ASOs designed by this module were tested *in vitro* and showed effectiveness in modulating IDO1 expression. However, the linear model has room for improvement. It achieved a Pearson correlation of only 0.72 for longer sequences, suggesting the need for more sophisticated models with nonlinear relationships or additional input features to enhance predictive accuracy. At the chemical engineering stage, ASO sequences are further refined to enhance their inhibitory activity while reducing potential cytotoxicity. ASOptimizer employs a deep graph neural network architecture known as edge-augmented graph transformer (EGT), which is adept at handling graph-related tasks. The EGT model is based on the molecular representations of ASOs, with learned embeddings for both atoms and bonds within the molecules. These embeddings are refined through multiple layers of the EGT model to predict the regulatory performance of the input ASO at the molecular level. Rather than solving a regression problem directly, the model was trained using a learning-to-rank approach to preserve the relative order of experimental knockdown efficacies among different ASOs. During training, pairs of ASOs were fed into the model, and it was optimized to correctly rank them according to their observed efficacy.

ASOptimizer presents a computational approach to design optimal ASOs with tailored chemical modifications. It integrates both sequence-specific and molecular-level optimizations to generate ASOs that exhibit improved regulatory functions and reduced toxicity profiles. It is a promising tool for advancing RNA-targeted therapies, especially those aiming to modulate IDO1 expression for cancer immunotherapy purposes. The research provides comprehensive evidence from *in vitro* experiments, confirming that optimized ASOs significantly inhibit IDO1 expression, thereby demonstrating their therapeutic potential in combatting cancer. This achievement not only underscores the power of deep learning technology in nucleic acid drug design but also provides a more advanced and practical tool for gene expression regulation research.

**Figure 1:**
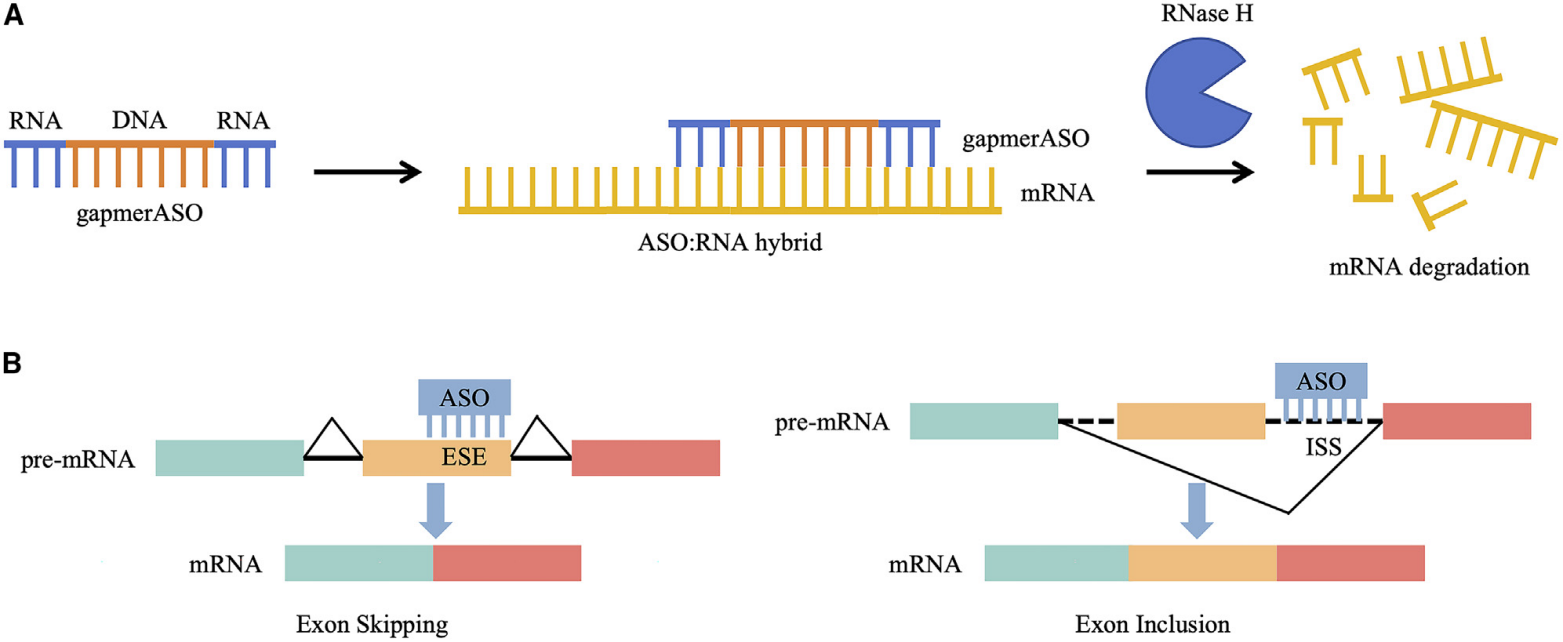
Overview of the action mechanisms of ASOs (A) Gapmer ASOs bind to their target mRNA to form DNA:RNA hybrids that recruit RNase H for the cleavage of mRNA. (B) The splicing of pre-mRNA is regulated by steric blocking.

Despite the significant achievements of ASOptimizer in IDO1 gene regulation, it is imperative to note the limitations imposed by the dataset, and further validation of the generalizability of ASOptimizer is warranted. Its applicability to other disease-related genes and complex disease models needs to be further validated. ASOs primarily regulate the function of target RNA through two mechanisms. The first is the degradation mechanism, in which ASO forms a heteroduplex with mature mRNA, leading to mRNA degradation through various cellular degradation pathways, thereby inhibiting gene expression (Figure 1A). The second is the steric blocking mechanism. After binding with RNA, ASO regulates processes such as transcription, splicing, and translation of RNA through steric hindrance effects, thereby inhibiting or promoting gene expression^9^ (Figure 1B). Ryu et al.’s study primarily focuses on RNase H-mediated ASOs within the degradation mechanism. The suitability of ASOptimizer for designing and optimizing ASOs based on the steric blocking mechanism requires further validation. Additionally, while the study makes breakthroughs in the chemical optimization of ASOs, it remains a critical issue to further enhance their stability and specific delivery *in vivo*.^10^ Moreover, the development of adaptive designs tailored to diverse disease states and individual variabilities, alongside the examination of potential adverse effects of ASOs within intricate pathological contexts, represent crucial directions that demand significant attention in future research endeavors.

In the coming years, the volume of experimental data pertaining to ASO therapy is expected to experience exponential growth. Therefore, the computational approach proposed by Ryu et al. is crucial for optimizing ASO therapy. Deep learning has demonstrated remarkable success in both small-molecule drug and protein sequence design and optimization. The successful implementation of ASOptimizer foreshadows the critical role that deep learning technology will play in nucleic acid therapy, represented by ASO drugs, ushering in a more efficient, cost-effective, and personalized era of nucleic acid drug development.
